# Mixed *Borrelia burgdorferi* and *Helicobacter pylori* Biofilms in Morgellons Disease Dermatological Specimens †

**DOI:** 10.3390/healthcare7020070

**Published:** 2019-05-17

**Authors:** Marianne J. Middelveen, Katherine R. Filush, Cheryl Bandoski, Rumanah S. Kasliwala, Anthony Melillo, Raphael B. Stricker, Eva Sapi

**Affiliations:** 1 Atkins Veterinary Services Ltd., Calgary, AB T3B 4C9, Canada; middel@telus.net; 2 Department of Biology and Environmental Science, University of New Haven, West Haven, CT 06516, USA; katherine.r.filush@gmail.com (K.R.F.); Cherylbandoski@gmail.com (C.B.); rumanahk@yahoo.com (R.S.K.); tmelillo8@gmail.com (A.M.); unh@evasapi.net (E.S.); 3 Union Square Medical Associates, San Francisco, CA 94108, USA

**Keywords:** Morgellons disease, *Borrelia burgdorferi*, *Helicobacter pylori*, biofilm, amyloidosis, tickborne disease, chronic infection, Lyme disease

## Abstract

Background: Morgellons disease (MD) is a dermopathy that is associated with tick-borne illness. It is characterized by spontaneously developing skin lesions containing embedded or projecting filaments, and patients may also experience symptoms resembling those of Lyme disease (LD) including musculoskeletal, neurological and cardiovascular manifestations. Various species of Borrelia and co-infecting pathogens have been detected in body fluids and tissue specimens from MD patients. We sought to investigate the coexistence of *Borrelia burgdorferi* (Bb) and *Helicobacter pylori* (Hp) in skin specimens from MD subjects, and to characterize their association with mixed amyloid biofilm development. Methods: Testing for Bb and Hp was performed on dermatological specimens from 14 MD patients using tissue culture, immunohistochemical (IHC) staining, polymerase chain reaction (PCR) testing, fluorescent in situ hybridization (FISH) and confocal microscopy. Markers for amyloid and biofilm formation were investigated using histochemical and IHC staining. Results: Bb and Hp were detected in dermatological tissue taken from MD lesions. Bb and Hp tended to co-localize in foci within the epithelial tissue. Skin sections exhibiting foci of co-infecting Bb and Hp contained amyloid markers including β-amyloid protein, thioflavin and phosphorylated tau. The biofilm marker alginate was also found in the sections. Conclusions: Mixed Bb and Hp biofilms containing β-amyloid and phosphorylated tau may play a role in the evolution of MD.

## 1. Introduction

Morgellons disease (MD) is a controversial illness characterized by spontaneously developing skin lesions that contain multicolored (mostly white, red, blue and black) filamentous inclusions and/or projections [1,2,3,4,5]. The dermopathy may be accompanied by symptoms of formication, and accordingly some health care practitioners claim that the condition is a variation of delusional parasitosis (DP) and that the filaments are implanted textile fibers [6,7].

In contrast, scientific studies indicate that the cutaneous filaments are physically and chemically consistent with human bioproducts. Histochemical studies revealed that they are hair-like collagen and keratin extrusions produced by keratinocytes and fibroblasts, respectively [5,8,9,10,11,12]. Furthermore, the blue color in fibers results from melanin pigmentation produced by melanocytes, thereby confirming a human bio-origin rather than a manufactured origin of the fibers [5,8,9,10,12]. Because MD fibers demonstrate apple green birefringence when stained with Congo Red, an amyloid component has been suggested in addition to collagen and keratin [10,12].

A spirochetal etiology for MD was considered because the dermopathy is accompanied by Lyme-like symptoms including musculoskeletal, neurological and cardiovascular manifestations [1,2,3,4,5,10]. Two independent cohort studies reported that almost all subjects with MD were diagnosed with LD and that there was a high incidence of infection with other tickborne pathogens [4,11]. MD occurs in a subset of approximately 6% of LD patients, and collectively the evidence suggests that MD results from a physiological response to infection [10,11,12,13].

*Borrelia* spirochetes that cause Lyme disease and “Lyme-like” illness including the *B. burgdorferi* (Bb) species Bb *sensu stricto* (Bbss) and Bb *sensu lato* (Bbsl) as well as Relapsing Fever *Borrelia* (RFB) have been detected in body fluids and/or tissue specimens from MD patients [10,12,14,15,16,17]. In addition to *Borrelia*, other pathogens such as *Helicobacter pylori* (Hp), *Treponema denticola*, *Bartonella henselae*, and *Rickettsia* spp. have been detected in MD patient specimens, suggesting that these pathogens could be co-involved in evolution of the dermopathy [10,12,17,18,19,20].

Bb and Hp have the capability to establish biofilms in vivo [21,22]. Borrelial lymphocytomas contain complex *Borrelia* aggregate structures exhibiting internal channels, surface protrusions and protective layers of mucopolysaccharide characteristic of biofilms [22]. Hp biofilms characterized by sessile bacteria surrounded by protective matrices have been detected in human gastric mucosal tissue. We hypothesized that *Borrelia* and/or Hp biofilm formation could be involved in MD development. We sought to gain a better understanding of MD by documenting the presence of Bb and Hp coinfection, and by identifying amyloid deposits and biofilm markers in skin exhibiting MD pathology, thereby exploring the possibility of mixed biofilm formation in vivo.

## 2. Materials and Methods

### 2.1. Specimen Collection

Dermatological specimens taken from lesions showing MD pathology were collected from 14 North American subjects who met the case definition for MD, as determined by a health care practitioner. The case definition used in this study required the presence of spontaneously developing skin lesions containing embedded or projecting red, white, blue or black filaments. Testing for LD or other pathogens prior to volunteering was not required for participation.

The MD skin specimens collected for study consisted of thickened callus material removed from lesions exhibiting embedded or projecting filaments. For comparative studies, normal healthy callused skin samples were collected from the feet of three healthy subjects, two subjects with LD but no MD, and one MD subject. Normal commercially available human skin (BioChain Institute, Newark, CA, USA) was included as a negative control for comparison purposes. MD dermatological specimens and foot callus specimens were stored as dried flakes, then forwarded to the University of New Haven Lyme Disease Research Laboratory in a blinded manner.

The study was conducted in accordance with the Declaration of Helsinki. Informed consent for study participation was obtained from MD subjects and control subjects in accordance with the specimen collection protocol approved by the Western Institutional Review Board (WIRB), Puyallup, WA, and consent to publish the study results was obtained from all subjects. The study was also approved by the University of New Haven IRB Committee as exempt under 45 CFR 46.101(b)(4). Subject identification, health status and demographic information were not provided to the research laboratory.

### 2.2. Culture of Bb and Hp

Skin specimens from MD subjects were cultured for Bb and Hp using a modification of the selective Hp culture system [23]. In brief, 400 mL of sterile water was mixed with 19 g of Columbia blood agar base (CM0331, Thermo Scientific, Waltham, MA, USA) and then autoclaved. After cooling to 50 °C, 35 mL of laked horse blood (SR0048, Thermo Scientific), the Hp antibiotic supplement (vancomycin 1%, trimethoprim 0.5%, cefsulodin 0.5% and amphotericin B 0.5%: SR0147, Thermo Scientific) and 100 mL of BSK-H complete medium with 6% rabbit serum (Sigma Aldrich, #B8291, St. Louis, MO, USA) were added and mixed well before pouring into sterile Petri dishes. The dishes were inoculated with scabs and incubated. Culture fluid was examined by darkfield microscopy for the presence of microorganisms. Culture smears were stained with either a standard Gram stain kit (Daylynn Biologicals, Calgary, AB, USA) to determine Gram reactivity [23], or with crystal violet (Daylynn Biologicals, Calgary, AB, USA) to visualize morphological features of the bacteria.

### 2.3. DNA Extraction

Dermatological specimens were homogenized by freezing in liquid nitrogen and grinding with a mortar and pestle. Specimens were resuspended in 180 µL of Buffer ATL (Qiagen Sciences, Germantown, MD, USA) with 20 µL of Proteinase K (Qiagen, Hilden, Germany), then incubated overnight at 56 °C with shaking. DNA was purified by standard phenol/chloroform extraction and alcohol precipitation the following day. Purified DNA was resuspended in 50–100 μL of 1× TE buffer at pH 8.0 (10 mM Tris, pH 8.0, and 1 mM EDTA).

### 2.4. Nested PCR

Nested PCR was performed to detect the presence of Bb and Hp DNA targets from purified DNA extracted from blinded MD and control specimens using “outer” primers for the first reaction and “inner” primers for the nested reaction. A volume of 10 µL of template DNA was used for the first reaction and 1 μL of PCR product from the first reaction was used as template for the second. Positive and negative controls were conducted alongside test specimens for each assay. The “No DNA” template was used as a negative control in all PCR reactions. As additional negative controls, the same PCR experiments were carried out on normal human skin samples. The positive control experiments used DNA extracted from low passage of Bb strain B-31 (ATCC #35210) and from frozen vials of Hp (ATCC #BAA-945). Primers used for the amplification of Bb and Hp were chosen from those used previously in the literature and they represented a mixture of conserved and polymorphic genes in order to define species and genotypes. PCR primers for Bb were: *pyrG*, *uvrA*, *fla* and *OspC* [24,25,26,27]. A summary of Bb PCR primers is shown in Table 1. PCR primers for Hp were: 16S rRNA, *hsp60*, *urea*, and 23S rRNA [28,29,30,31]. A summary of Hp PCR primers is shown in Table 2.

PCR reactions were performed using a final volume of 50 µL and final concentrations of 1× Buffer B (Promega), 1.5 mM MgCl_2_, 200 µM dNTP mix, 0.2 µM of each primer, and 2.5 U Taq polymerase (Invitrogen, Carlsbad, CA, USA). Cycling parameters were: 95 °C for 5 min followed by 35 cycles of denaturation at 95 °C for 1 min, annealing for 1 min (temperature based on the primer set used), and extension at 72 °C for 1 min, with a final extension step at 72 °C for 5 min. PCR products were visualized on 1–1.5% agarose gels stained with ethidium bromide.

Sanger sequencing was used to confirm that the target DNA detected was correct. PCR products were purified using the QIAquick Gel Extraction kit (Qiagen) according to the manufacturer’s instructions. The eluates were sequenced in both directions by Eurofins MWG Operon DNA sequencing services (Eurofins Scientific, Louisville, KY, USA). The sequences were compared to those in the National Center for Biotechnology Information (NCBI, Bethesda, MD, USA) GenBank database using Basic Local Alignment Search Tool (BLAST) analysis. Only PCR amplicons confirmed as correct by BLAST analysis were interpreted as positive.

### 2.5. Preparation for Histochemical Staining

MD dermatological specimens and normal human skin controls were formalin-fixed, paraffin-embedded and sectioned. Sections were deparaffinized, rehydrated, blocked with a 1:100 dilution of goat serum (Thermo Scientific) in 1× PBS phosphate buffered saline (PBS, Sigma, St. Louis, MO, USA) for 30 min at room temperature in a humidified chamber, then washed twice in 1× PBS (Sigma) for 5 min followed by distilled water for 5 min.

### 2.6. Dual Bb and Alginate Immunohistochemical (IHC) Staining

Blocked, washed sections were reacted with a 1:500 dilution (1× PBS/1%BSA) of primary anti-alginate antibody (kindly provided by Dr. Gerald Pier, Harvard Medical School, Boston, MA, USA), placed in a humidified chamber, incubated overnight (minimum of 16 h) at 4 °C, followed by washing in PBS as described in the preparation above. The sections were then reacted with 1:200 dilution of a secondary anti-rabbit antibody with a fluorescent red tag (594 nm, Thermo Scientific) for 1 h in a humidified chamber at room temperature, then washed in PBS as described above. Sections were then stained with a 1:10 dilution of mouse monoclonal antibody specifically targeting Bb *sensu stricto* strains (MA-1-7006, Thermo Scientific), incubated overnight (minimum of 16 h) at 4 °C, then washed as described above and further incubated with a 1:200 dilution (1× PBS/1%BSA) of goat anti-mouse secondary antibody labeled with DyLight 488 fluorescent dye in a humidified chamber at room temperature. The sections were washed again as described above and counterstained with Sudan Black (Sigma) for 20 min, then stained with 4’,6-diamidino-2-phenylindole (DAPI) for 90 s. Sections were washed, allowed to dry, then mounted with PermaFluor (Thermo Scientific). Images were captured using a Leica DM2500 fluorescent microscope at 200× and 400× magnification. As negative controls, commercially available human foreskin tissue sections and healthy human skin sections (Biomax, HuFPT136) were stained following the same procedure described above. Additional negative control experiments omitting the primary antibody and using a non-specific mouse IgG1 isotype (MA1-10406, Invitrogen) were also performed to confirm the specificity of the antibodies.

### 2.7. Hp IHC Staining

Consecutive sections of Bb and alginate positive blocks were further analyzed for the presence of Hp. Deparaffinized and washed sections were blocked with goat serum as described above and then incubated for 1 h with a 1:50 dilution of a fluorescently-labeled polyclonal rabbit anti-*Helicobacter pylori* antibody targeting ATCC 43504 whole Hp cells (PA1-73161, Thermo Scientific) for 1 h in a humidified chamber at room temperature. Sections were then washed, counterstained with Sudan Black, mounted and imaged as described above.

### 2.8. Dual β-Amyloid or Phospho-Tau IHC Staining and Bb or Hp IHC Staining

Staining for β-amyloid proteins and phosphorylated tau was performed on consecutive sections from blocks that were positive for Bb and Hp [32]. Sections were processed and washed as described above, followed by incubation with a 1:200 dilution of either anti-β-amyloid antibody-1 (ABA-1) (MA1-34553, Thermo Fisher Scientific), or anti-β-amyloid antibody-2 (ABA-2) (M 0872, Daco Scientific Ltd., Reading, UK), following previously published IHC protocols [32]. Alternatively, sections positive for Bb and Hp were incubated with 1:200 dilutions of anti-phosho-tau antibody (MN1020, Thermo Scientific). As a negative IHC control, a non-specific mouse IgG1 isotype (MA1-10406, Invitrogen) was used in place of the primary antibodies to confirm the antibody specificity of this assay.

### 2.9. Thioflavin Histochemical Staining

Deparaffinized, rehydrated, formalin-fixed, sections were stained with 1% Thioflavin S (Santa Cruz Biotech, Santa Cruz, CA, USA) solution in 80% ethanol and incubated for 15 min at room temperature. Sections were washed in an 80% ethanol bath, then a 70% ethanol bath for 1 min each, followed by washing in double distilled water twice for 5 min each time. Sections were then mounted and imaged as described above.

### 2.10. FISH Testing

FISH was adapted from previously validated probes for both Bb and Hp [22,33,34,35]. The sequences of the Bb and Hp DNA probes are listed in Table 3. Slides of paraffin-embedded tissue sections were first deparaffinized by heating on a slide warmer for 40 min at 45 °C, then immersed in 100% xylene for 5 min three times. The tissue sections were rehydrated in series of graded alcohols (100%, 90% and 70%) and washed in PBS for 5 min followed by washing with distilled water for 15 min. The sections were then treated with 4% sodium borohydride (Sigma Aldrich) for 20 min on ice. The tissues were then digested with pre-warmed proteinase K solution (20 µg/mL in 50mM Tris) for 10 min at 37 °C and re-fixed with 4% paraformaldehyde for 10 min at room temperature. The slides were then denatured using pre-heated denaturing buffer (70% v/v formamide, 2× SSC and 0.1 mM EDTA, pH 7, Thermo Fisher, Scientific) at 70 °C followed by prehybridization for 4 h in hybridization buffer (50% v/v formamide (Sigma), 10% w/v dextran sulfate (Sigma), 1% v/v Triton X-100 (Sigma), 2× SSC, (Sigma) pH 7.0, and 2 ng of salmon sperm DNA (Sigma) in an incubator at 48 °C.

Hybridization was achieved by reacting the slides with the probe at 48 °C for 18 h in the dark. Post-hybridization, slides were washed three times with 2× SSC for 3 min each at room temperature followed by 5 washes each for 20 min in 0.1× SSC at 42 °C and a final wash in 2× SSC at room temperature. Finally, slides were blocked with freshly made blocking solution (3% w/v BSA (Fisher) in 4× SSC, 0.1% v/v Triton X-100) for 30 min and washed with wash solution (4× SSC, 0.1 % v/v Triton X-100) for 3 min at room temperature. All steps were repeated with several controls including (1) 100 ng of negative control random oligonucleotide, (2) 200 ng of unlabeled competing oligonucleotide present during the hybridization and (3) following a DNase treatment of the sections before the hybridization step to digest all genomic DNA (100 µg/mL for 60 min at 37 °C). All slides were then analyzed by fluorescent microscopy and images captured as described previously.

### 2.11. Confocal Microscopy

Dual IHC staining for Bb and Hp in MD skin sections was performed as described above. Deparaffinized and washed sections were stained with mouse monoclonal antibody specifically targeting Bb *sensu stricto* strains (MA-1-7006, Thermo Scientific) followed by staining with goat anti-mouse secondary antibody labeled with DyLight 488 fluorescent dye as described above. The sections were then washed, blocked with goat serum and incubated with fluorescently labeled polyclonal rabbit antibody targeting whole Hp cells (PA1-73161, Thermo Scientific) in a humidified chamber at room temperature. Sections were then washed and counterstained with Sudan Black. Confocal microscopy with a Leica DMI6000 was performed in order to increase optical resolution and contrast so that spatial distribution of mixed biofilm structures could be observed in 3 dimensions. Z-stacks were generated using Image J software (Wayne Rasband, NIH, Bethesda, MD, USA).

## 3. Results

### 3.1. Culture of Bb and Hp

**Figure 1:**
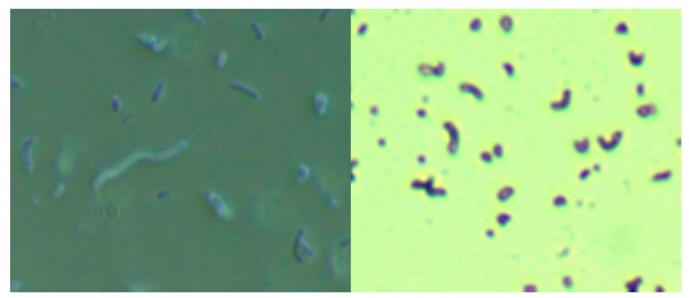
Culture of *Borrelia burgdorferi* and *Helicobacter pylori* from a Morgellons disease (MD) skin sample in modified Hp medium. Left Panel: Darkfield microscopy showing larger *Borrelia* spirochete surrounded by smaller organisms. Right Panel: Crystal violet stain showing comma-shaped Gram-negative organisms consistent with *Helicobacter pylori*. Both images were taken at 1000× magnification.

The culture of skin specimens from two MD subjects in modified selective Hp medium revealed spirochetes and Gram-negative comma-shaped organisms, as shown by darkfield microscopy and crystal violet staining in Figure 1. The spirochetes tested positive using monoclonal anti-Borrelia antibody (data not shown). Further molecular testing for Bb and Hp in skin samples is outlined below.

### 3.2. Nested PCR

Using nested PCR technology, specific DNA targets for Bb were detected in dermatological specimens from 10/14 (71%) MD subjects; specific Hp DNA targets were detected in dermatological specimens from 12/14 (86%) MD subjects; specific DNA targets for both Bb and Hp were detected in dermatological specimens from 8/14 (57%) MD subjects; and 0/14 (0%) were negative for both Bb and Hp. All normal skin samples from healthy controls and Lyme and MD subjects tested negatively for both Bb and Hp, as did the purchased normal human skin. The results are summarized in Table 4.

BLAST analysis of all Bb amplicon sequences most closely matched Bbss, and BLAST analysis of Hp sequences matched several different Hp species. Table 5 summarizes the results of Bb BLAST analyses, and Table 6 summarizes the results of Hp BLAST analyses. All sequence data and BLAST analyses are available in the supplemental information section.

### 3.3. Histochemical Staining

Bb and Hp positive skin specimens from 6/14 of the above subjects were tested in further studies using histochemical staining and FISH. Sections of normal human skin were used as negative controls, while brain from a mouse infected with Bbss and bacterial smears of Bbss B-31 and Hp culture (ATCC #BAA-945) were used as positive controls. All negative controls yielded negative results and all positive controls yielded positive results (data not shown). The results of histochemical staining are summarized in Table 7.

### 3.4. Bbss IHC Staining

**Figure 2:**
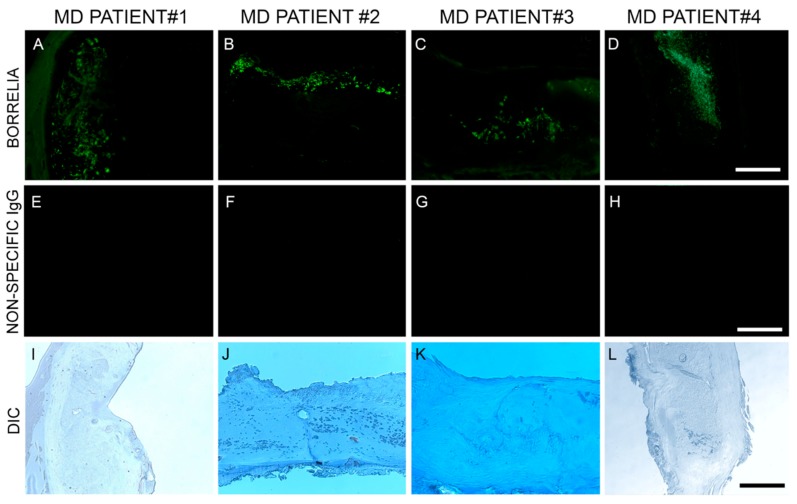
Representative immunohistochemical (IHC) images of *Borrelia burgdorferi* aggregates in skin sections of four MD subjects. IHC detection was performed as described in the Methods section. Panels labeled A–D show *Borrelia burgdorferi* aggregates in skin sections treated with anti-*Borrelia* monoclonal antibody (green). Panels labeled E–H show same sections treated with non-specific IgG antibody. Panels labeled I–L show the same sections under brightfield illumination with differential interference contrast (DIC) microscopy. Images were taken at 200× magnification. Scale bar = 100 µm.

Anti-Bb monoclonal antibody reacted positively to sections from 6/6 patient specimens. Bb spirochetes were visible individually with characteristic spirochetal morphology or adhering together in groups. See Figure 2.

### 3.5. Alginate IHC Staining

**Figure 3:**
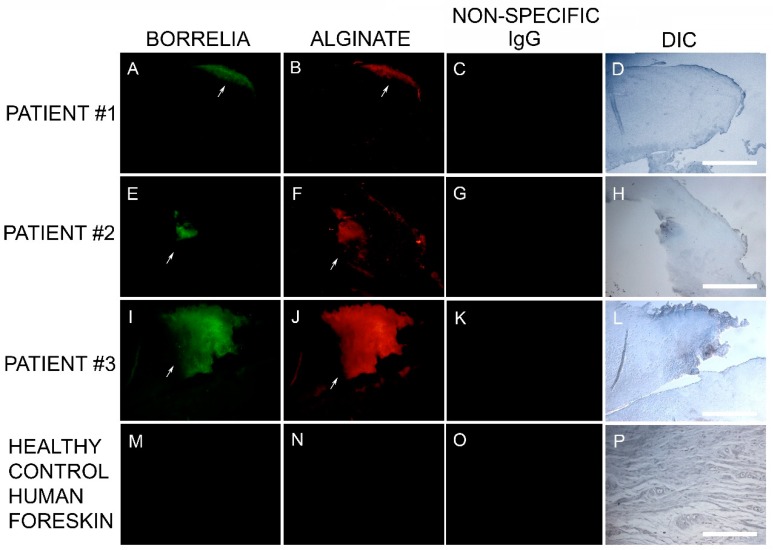
Representative IHC images showing *Borrelia*- and alginate-positive aggregates in MD skin sections from three subjects. IHC detection was performed as described in the Methods section. Panels A, E, I and M show skin sections treated with anti-*Borrelia* monoclonal antibody (green). Panels B, F, J and N show skin sections treated with anti-alginate antibody (red). Panels C, G, K and O show control skin sections treated with non-specific IgG. Panels D, H, L and P show sections imaged with DIC. All images were taken at 200× magnification. Scale bar = 100 µm.

The Bb aggregate formations in MD tissue resembled in vitro Bb biofilm formations that we had previously observed in our laboratory. Thus, we hypothesized that biofilm formation in vivo might be occurring in MD tissue. This finding led us to test for the presence of alginate in Bb-aggregate formations. Dual reactivity of anti-Bb and anti-alginate antibodies to MD skin sections was noted in sections from 6/6 subjects. The positive IHC staining for Bb and alginate antigens occurred in aggregate formations that were co-localized. This demonstration of Bb/alginate aggregate formation suggested Bb biofilm formation occurred in vivo—within the skin from MD subjects that demonstrated dermopathy. Non-specific isotype IgG controls were used instead of the primary antibodies as IHC negative controls to confirm the specificity of *Borrelia* and alginate-specific antibodies. Representative images can be seen in Figure 3.

### 3.6. Hp IHC Staining

**Figure 4:**
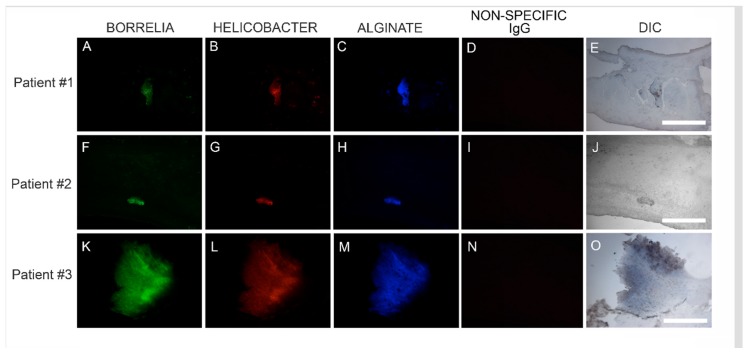
Representative IHC images showing biofilm aggregates in MD skin sections stained for *Borrelia*, *Helicobacter* and alginate. IHC detection was performed as described in the Methods section. Panels A, F and K show skin sections treated with anti-*Borrelia* monoclonal antibody (green). Panels B, G and L show skin sections treated with anti-*Helicobacter* antibody (red). Panels C, H and M show skin sections treated with anti-alginate antibody (blue). Panels D, I and N show negative control sections treated with non-specific IgG. Panels E, J and O show sections imaged with DIC. Images were taken at 200× magnification. Scale bar = 100 µm.

We subsequently sought to determine the location of Hp infection in relation to Bb/alginate aggregates using Hp-specific IHC staining on consecutive sections of Bb/alginate positive specimens. Positive reactivity of Hp antibodies co-localized with Bb/alginate aggregates in 6/6 patient specimens. This demonstration of Bb/Hp/alginate aggregate formation suggested mixed Bb/Hp biofilm formation occurred in vivo—within the skin from MD subjects that demonstrated dermopathy. Representative images are shown in Figure 4.

### 3.7. Bb and Hp FISH

**Figure 5:**
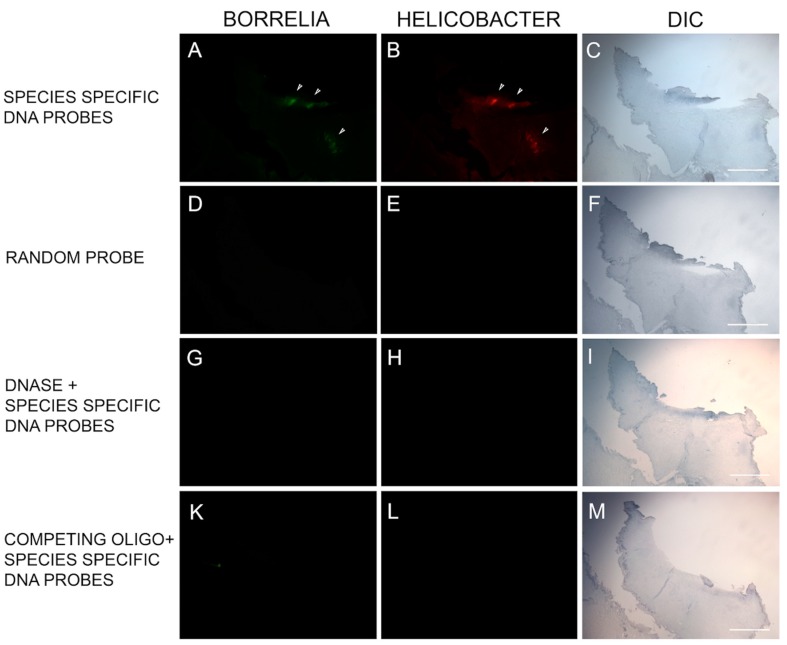
Representative images of the fluorescent in situ hybridization (FISH) reactivity of *Helicobacter* and *Borrelia* DNA within an MD skin section. FISH detection was performed as described in the Methods section. Panel A shows reactivity of MD skin sections using a 16s rDNA probe specific for *Borrelia burgdorferi* (green). Panel B shows reactivity of MD skin sections using a 16s rDNA *Helicobacter pylori* probe (red). Panels D, E, G, H, K and L show various controls as noted. White arrows indicate positive staining. Panels C, F, I and M show the section imaged with DIC. Images were taken at 100× magnification. Scale bar = 200 µm.

DNA/DNA FISH was performed on specimens from six subjects to provide corroborative evidence using an alternative methodology. The Bb-specific DNA probe and the Hp-specific DNA probe bound to their respective DNA targets in 6/6 of the MD skin sections tested. Mixed Bb and Hp DNA in aggregate formations was detected in 6/6 specimens, and one specimen had DNA from both organisms without overlapping Bb and Hp DNA targets. See Figure 5.

### 3.8. Amyloid Detection

Theorizing that amyloid proteins could provide structural support for biofilm formation, MD skin sections from blocks known to contain foci of Bb and Hp were screened for possible amyloidosis using histochemical and IHC staining specific for Thioflavin S, β-amyloid protein, and phospho-tau. The results are summarized in Table 7.

**Figure 6:**
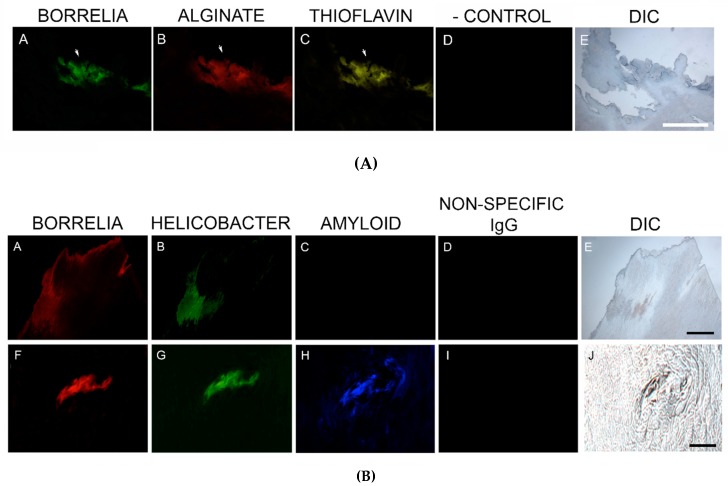
(**A**) Representative images of aggregate biofilm structures in MD skin sections demonstrating positive staining for *Borrelia*, alginate and Thioflavin S. IHC detection and Thioflavin S staining was performed as described in the Methods section. Panels A and B show positive IHC staining for *Borrelia* (green) and alginate (red) in aggregate structures. Panel C shows fluorescent staining using Thioflavin S (yellow) on *Borrelia* and alginate-positive aggregate structures. Panel D shows negative staining with non-specific IgG. Panel E shows the section imaged with DIC. (**B**) Representative IHC images of aggregate biofilm structures demonstrating antibody reactivity with *Borrelia*, *Helicobacter* and amyloid in MD skin sections. IHC detection was performed as described in the Methods section. Skin sections were treated with two different fluorescent anti-amyloid antibodies, ABA-1 and ABA-2. Panels A and F show positive fluorescent staining for *Borrelia* (red), and panels B and G show positive fluorescent staining for *Helicobacter* (green) in aggregate structures. Panel C shows negative staining using ABA-1, while Panel H shows positive staining using ABA-2 (blue) on *Borrelia* and *Helicobacter*-positive aggregate structures. Panels D and I show negative staining with non-specific IgG. Panels E and J show sections imaged with DIC. All images were taken at 200× magnification. Scale bar = 100 µm.

Thioflavin S staining was performed on 2/6 patient specimens. The staining of these two specimens was positive, and staining co-localized with foci of infection. Thus, Bb/Hp comingled formations suggestive of biofilm formation were also positive for Thioflavin S staining. See Figure 6A.

Confirmatory staining for amyloid in skin specimens that were known to contain foci of Hp and Bb was performed using IHC staining with two different antibodies specifically targeting β-amyloid proteins, on sections from the blocks known to contain foci of Bb and Hp. Staining for β-amyloid protein with ABA-1 antibody was negative in all six specimens. In contrast, MD sections stained with ABA-2 antibody revealed positive results in all six specimens, indicating that β-amyloid protein was indeed present in MD specimens. See Figure 6B.

**Figure 7:**
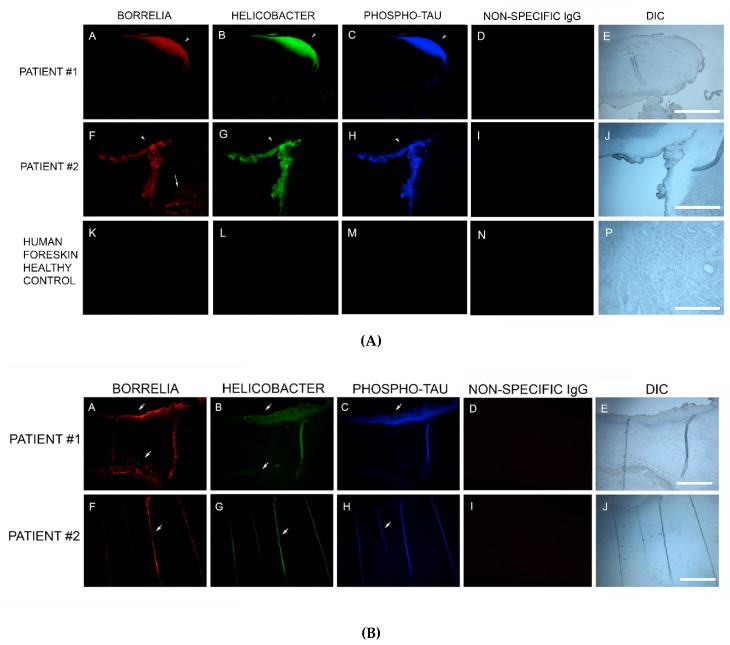
(**A**) Representative IHC images of phospho-tau reactivity co-localizing with Borrelia and Helicobacter in aggregate biofilm structures of MD skin sections from two subjects (A–D and E–H). IHC detection was performed as described in the Methods section. Panels A and E demonstrate positive reactivity with anti-Borrelia burgdorferi antibody (red). Panels B and F demonstrate positive reactivity with anti-Helicobacter antibody (green). Panels C and G demonstrate positive reactivity with anti-phospho-tau antibody (blue). Panels I, J and K demonstrate lack of reactivity with non-specific antibody. Panels D, H and L show sections imaged with DIC. (**B**) Representative IHC images of phospho-tau reactivity co-localizing with Borrelia and Helicobacter in fibers of MD skin sections from two subjects (A–D and E–H). IHC detection was performed as described in the Methods section. Panels A and E show reactivity with anti-Borrelia antibody (red). Panels B and F show reactivity with anti-Helicobacter antibody (green). Panels C and G show reactivity with anti-phospho-tau antibody (blue). Panels D and H show sections imaged with DIC. All images were taken at 200× magnification.

Sections from 6/6 MD specimens were tested for the presence of phospho-tau, using IHC staining with an antibody reactive to phospho-tau, along with staining with antibodies specific for Bb and Hp. Anti-phospho-tau antibody reacted positively to all six MD skin sections tested. The areas that were reactive co-localized with positive Bb and Hp staining. This demonstrated that Bb/Hp comingled formations were positive for the presence of phospho-tau. See Figure 7A.

In addition, IHC staining with antibodies specific for Bb, Hp and phospho-tau was performed on two callus specimens containing the filamentous inclusions characteristic of MD. The antibodies reacted with the filamentous inclusions. This finding suggests that Bb, Hp and phospho-tau could play a role in MD fiber evolution. See Figure 7B.

### 3.9. Confocal Microscopy

**Figure 8:**
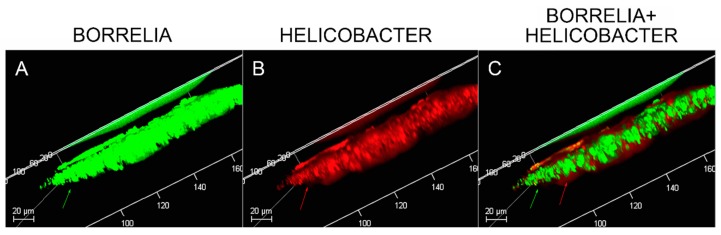
Confocal microscopy of MD skin sections. Confocal microscopy was performed as described in the Methods section. Panel A, staining for *Borrelia* alone (green). Panel B, staining for *Helicobacter* alone (red). Panel C, staining for both *Borrelia* and *Helicobacter*. Note, *Helicobacter* localization occurred mainly on the external part and *Borrelia* localization occurred mainly on the internal part of the section. All images were taken at 200× magnification.

MD tissues that contained Bb/Hp-positive biofilm structures were scanned using confocal microscopy to obtain a three-dimensional analysis of the mixed biofilms. Z-stacks acquired through confocal microscopy were analyzed for the spatial distribution of the two different pathogenic species. Figure 8 shows a representative image of the confocal microscopy scanning, demonstrating that there is a very specific distribution for Bb and Hp cells in biofilms. The distribution shows that Hp cells localized mainly on the outside of the structure, while Bb cells concentrated in the middle of the biofilm.

## 4. Discussion

In this study, Bb/Hp dual infection was detected in MD skin sections using tissue culture, PCR technology, IHC staining, FISH testing and confocal microscopy. The PCR data from this study revealed that callus material from 14 MD subjects tested positive for at least one sequence from either Bb or Hp, and eight subjects tested positive for at least one sequence from both bacteria. This finding verified that Bb and Hp are pathogens that can be present in MD skin sections. Given the fact that both Bb and Hp infections can cause illness in humans, including dermopathy, the presence of Bb and Hp in skin demonstrating MD pathology strongly suggests that these organisms could jointly contribute to MD evolution. IHC staining and FISH testing for Bb/Hp overlapped on consecutive sections, revealing that these pathogenic organisms co-localize in aggregates consistent with biofilms.

Species of *Borrelia* from both the LD and RFB groups have been repeatedly detected in MD tissues, suggesting a causal relationship [10,12,16]. The genus *Borrelia* comprises at least 52 species from the LD group (Bbss and Bbsl) and the RFB group—members of these species being the causative agents of Lyme disease and relapsing fever, respectively [37,38,39,40,41]. Lyme disease is a systemic infection involving multiple organs including joints, the heart, and the central nervous system [42]. RFB produces systemic infection with cyclical fevers, flu-like symptoms and possible central nervous system involvement, especially in immunocompromised patients [43]. Although dermatological manifestations are not well described for RFB, various skin ailments are associated with LD. There are three skin conditions commonly attributed to Bb: erythema migrans, lymphocytoma, and acrodermatitis chronica atrophicans. [44]. In addition, other dermatological conditions have been attributed to Bb infection, including morphea, lichenoid genital lesions, B-cell lymphoma, dermatomyositis and cutaneous lymphoid hyperplasia [44,45,46,47].

Hp has been detected less frequently in MD skin sections [10,12,18,19]. Hp is a bacterium commonly encountered in humans, with as many as 90% of the population estimated to be colonized [48,49]. It is primarily associated with gastrointestinal inflammatory and ulcerative diseases, and it is the primary etiologic agent in chronic active gastritis, peptic ulcer disease, gastric adenocarcinoma, and mucosa-associated lymphoid malignancy [49]. Studies have linked Hp infection to gastric cancer in humans, and infection is classified as carcinogenic [50]. Hp infection may also be associated with extra-intestinal pathology including endocrine and metabolic disorders, immune dysfunction, rheumatological conditions and autoimmune diseases [49,51,52,53]. Hp may also play an etiological role in various skin disorders including rosacea, psoriasis, chronic urticaria, Schönlein–Henoch purpura, and alopecia areata [51,53,54].

Biofilms are bacterial communities formed by many bacterial species in response to environments that are adversarial, including factors such as high pH, suboptimal oxygen levels, host immune attack or antibiotics [55]. The formation of biofilm is a collective bacterial behavior. Bacteria use quorum sensing to regulate the gene expression required for collective behaviors, and these processes require a group-level response to extracellular signaling molecules called autoinducers that are produced and secreted by the bacteria [56]. Biofilms can form on abiotic and biotic surfaces, including mucosal surfaces and on indwelling medical devices [55]. Bacterial biofilms can form in vivo in human beings, resulting from infections associated with chronic conditions such as bacterial endocarditis, cystic fibrosis, human gastric mucosal lesions and struvite nephrolithiasis [57,58]. Biofilms along with resultant immune responses are responsible for the development of chronic recalcitrant slowly-healing wound conditions such as venous leg ulcers and diabetic foot ulcers and are acknowledged contributors to antibiotic resistance [59,60,61,62]. Like other chronic skin ulcerations, MD lesions are slow to heal and the establishment of Bb/Hp mixed biofilms in MD cutaneous tissue would help to explain the chronicity and recalcitrance of MD lesions.

Bb and Hp can independently form biofilms both in vitro and in vivo [21,22,58,60,62,63]. The formation of biofilms in vitro enabled the establishment of antibiotic resistant Bb cultures [60,64]. Although chronic LD is a controversial topic, persistent Bb infection despite antibiotics was reported in a cohort of patients with Lyme disease that included MD subjects, and viable Bb spirochetes were recovered by culture from body fluids and tissue specimens from these subjects [65]. Antimicrobial resistance of Hp is a major concern, with 30% of clinical isolates estimated to be clarithromycin resistant [49]. Although the exact mechanism of resistance has not been elucidated, biofilms may contribute to the establishment of recalcitrant infections caused by both Bb and Hp [49,60,64].

Callus sections from the six subjects with aggregates positively staining for Bb and Hp also stained positive for alginate. The presence of alginate in aggregate structures implies that mixed Bb/Hp biofilms contribute to MD pathology. Biofilm microbial communities reside in a complex self-produced exopolymeric matrix (EPM) comprised of lipids, amyloid-like proteins, exopolysaccharides, environmental DNA, and other substances [55,66]. The EPM serves numerous functions: structural support, protection from the external environment, gene regulation and nutrient absorption [36,55,59,60,66]. The mucopolysaccharide component of the EPM is a protective barrier against hostile external factors, and alginate is a key mucopolysaccharide component of many bacterial biofilms [22,67,68,69]. Alginate expression occurs in *Leptospira* and *Borrelia* spirochetal biofilms, as well as in the biofilms formed by other bacterial species, such as those produced by *Psuedomonas aeruginosa* in the lungs of cystic fibrosis patients [22,58,67,68,69,70].

In vitro, Bb biofilms express alginate in the EPM and exhibit elaborate structures with channels and protrusions [22,60,71]. The Bb biofilms found in borrelial lymphocytomas also feature channels and protrusions that are comparable to those of Bb biofilms formed in vitro [22]. In borrelial lymphocytoma tissue sections, alginate was associated with Bb aggregates but not isolated spirochetes, a finding that is consistent with biofilm formation. Although the exact mechanism of alginate production by *Borrelia* spp. is not yet understood, an alternate method of biosynthesis involving enzymes of the Entner–Doudoroff pathway has been proposed [60]. Alternate pathways were not tested or confirmed in the current study. Hypothetically, Hp biofilms could also express alginate as a component of their EPM [49]. All MD calluses that demonstrated Bb/Hp aggregates were stained for alginate. Positive staining overlapped with Bb/Hp aggregates, providing convincing evidence that mixed biofilm formation occurred in vivo within calluses demonstrating MD pathology. Although alginate containing Bb/Hp aggregates were detected in MD tissue, further research is needed to determine whether Bb alone, Hp alone, or both Bb and Hp contributed towards generation of the alginate layer. Regardless of the specific origin of alginate, the presence of alginate co-localizing with Bb/Hp aggregates on MD skin tissue sections is indicative of mixed bacterial biofilm.

Confocal microscopy of MD callus tissue revealed that Bb and Hp distribution within Bb/Hp biofilm structures was species specific. Hp cells localized more superficially than Bb cells, while Bb cells localized mostly in the interior. Specific distribution suggests that these bacteria have specific functional roles within the structure that may be synergistic. Mixed bacterial infections have been reported to act in a synergistic manner, and mixed species bacterial biofilms are found in human oral cavities and have been shown to have a high degree of resistance to antibiotic treatment [72,73]. Simultaneous cutaneous experimental infection of laboratory animals with streptococci and *Staphylococcus aureus* induced a necrotizing condition that did not occur after experimental infection with either organism alone [72]. Synergistic interactions between bacteria in mixtures—*Acinetobacter baumanni* combined with *Streptophomonas maltophila*, and *Pseudomonas aeruginosa* with *S. maltophila*—increased biofilm formation from 20-fold to as much as 102-fold when compared to biofilm formation in single-species cultures. The synergistic augmentation of biofilm formation was noted even when one of the organisms in the mixture inherently lacked the ability to form biofilms [74].

In addition to enhanced biofilm formation, other synergistic effects between mixed bacteria occur. For example, *Haemophilus influenzae* expresses its virulence factor type IV pili in a mixed-species biofilm with *Streptococcus pneumoniae* [74]. Synergistic enhancement of biofilm production could also involve a variety of mechanisms: one species of bacteria may provide surface attachment for another species; or production of secreted factors such as quorum-sensing molecules may be increased; or production of other bacterial cellular products such as those that influence gene expression, metabolic interaction, physical contact, and synthesis of antimicrobial secretory exoproducts may be increased [74]. Synergy in some mixed bacterial biofilms is dependent upon cell–cell contact rather than the effects of soluble bacterial products [75]. As other pathogens such as *Treponema denticola*, *Bartonella henselae*, and *Rickettsia* spp. have been detected in MD skin sections, they too could be co-involved in mixed biofilm formation [10,12,17,18,19,20]. It is possible that these and other yet undetected pathogens in MD skin could be present in mixed MD biofilms, and the constellation of pathogens contributing to MD evolution may differ from patient to patient. The synergistic effects of Bb and Hp in MD biofilms, their species-specific contributions towards establishing the biofilm structure and their joint role in MD evolution remain to be elucidated.

MD callus tissue sections were screened for the presence of amyloid deposits by staining with a general amyloid stain, Thioflavin S, that enhances the fluorescence of laminated β-sheets [76], followed by more targeted detection using IHC staining with antibodies specific for amyloid-β. Amyloids are abnormal fibrous, extracellular, insoluble, proteinaceous deposits, predominantly composed of β-sheet structures in a characteristic cross-β conformation [77]. There are 20 different plasma proteins that can form amyloid aggregates, and despite differences in their amino acid sequences, all have precursor molecules capable of forming aggregates and all have a β-strand polypeptide backbone capable of assembly into β-sheets [77].

Degenerative diseases such as Alzheimer’s disease (AD), prion-associated spongiform encephalopathies, type II diabetes, transthyretin amyloidosis, and Parkinson’s disease are associated with accumulation of insoluble misfolded protein aggregates, such as amyloid and tau aggregates, in afflicted tissues [77,78,79,80]. AD brains are distinguished by the formation of senile plaques composed of extracellular β-amyloid protein (β-A4) deposits and/or filamentous curli fiber aggregates and the formation of neurofibrillary tangles that are composed of paired helical filaments of insoluble highly phosphorylated tau proteins and associated lipids [32,81]. The AD amyloid β-A4 is a 4-KDa protein resulting from the cleavage of β-amyloid precursor protein (βAPP) [81,82]. Under normal circumstances, βAPP is a transmembrane protein associated with neuronal development, neurite outgrowth, and axonal transport [81].

Pathological argyrophillic filaments, histochemically consistent with amyloids, accumulate in AD brain lesions, but may also accumulate in other diseased organs such as pancreas, liver, ovaries, testes and thyroid [32]. Amyloid deposition resulting from abnormal protein folding and assembly can occur in other tissues including skin [83,84,85]. In primary cutaneous amyloidosis, the amyloid deposits are restricted to skin, while in secondary cutaneous amyloidosis (associated with systemic amyloidosis), the amyloid deposits are located in skin and other organs [85,86].

Amyloid fibrils are associated with both Hp and Bb infections, serving as structural components of biofilms [83,84,87]. Pathology like that of AD can result from spirochetal infection. Furthermore, protein accumulation resembling that of AD occurs in patients with tertiary syphilitic dementia paralytica. [82] Bb expresses peptides with sequences capable of forming polymers in a β-sheet structure analogous to amyloid fibrils associated with human degenerative diseases [88]. Aggregate deposits resembling those seen in AD form in mammalian glial and neuronal cell cultures after exposure to Bb spirochetes. In addition, βAPP and phospho-tau levels in cell cultures increased after the introduction of spirochetes or borrelial lipopolysaccharides [82]. Our study established that MD specimens stained positively for the presence of β-amyloid and positive staining overlapped with Bb/Hp aggregates. Amyloids may therefore have a functional role in establishing and maintaining mixed Bb/Hp biofilm structures in MD skin and consequently in the evolution of MD.

Although amyloid fiber formation is largely considered to be due to aberrant protein folding, functional amyloids have been identified that provide organisms with beneficial properties [77,89,90]. These functional amyloids encompass a wide range of sources including a mammalian protein constituent of melanosomes called Pmel17, bacterial curli fibers and fungal hydrophobins [77,89,90]. Curli are functional amyloid fibers produced by Gram-negative bacteria such as *Escherichia coli* that serve as protein scaffolds within the EPM of biofilms [91]. Curli fimbriae are biophysically prototypical amyloid fibers. They are ordered protease-resistant β-sheet-rich fibers that bind with amyloid-specific dyes such as Congo Red or Thioflavin T [90].

It is hypothesized that bacteria-generated amyloids along with other bacterial secretory products may contribute to the pathology and evolution of amyloidogenic progressive immunological and neurological disorders [90]. Bacterial amyloids could activate neuropathogenic signals promoting human-generated amyloid aggregation and inflammatory degeneration characteristic of degenerative neurological diseases such as AD [90]. Bacteria- and host-derived amyloid-β are components of senile plaques and βAPP and/or βAPP-like amyloidogenic proteins are associated with spirochetes [92]. Hypothetically, abnormal human cutaneous amyloid deposits could be functionally beneficial to bacteria, perhaps providing a scaffold for biofilm formation or providing a protective barrier for bacteria in a hostile environment. The MD specimens in this study stained positively with only one of the two anti β-amyloid antibodies. The antibodies that we used were anti-human β-amyloid antibodies but targeted to different epitopes. The crossreactivity of the anti-human β-amyloid antibodies with Bb and/or Hp-generated amyloid is not known. It is possible that the amyloidosis we observed is bacteria-generated, rather than human-generated, or possibly a combination of bacteria- and human-generated amyloid folded protein, providing a possible explanation as to why MD Bb/Hp aggregates were reactive to only one antibody.

MD callus tissue sections also stained specifically for the presence of tau protein using IHC staining. Although the “amyloid hypothesis” that β-amyloid aggregation is the primary cause of AD was the predominant theory among AD researchers, new evidence suggests that AD arises by impairment of βAPP metabolism via tau pathology [81]. Tau protein is a microtubule-associated protein serving a functional role in promoting microtubule polymerization and stabilization, controlled by kinase regulated phosphorylation [81,93,94,95]. Six tau isoforms resulting from mRNA alternative splicing are expressed in adult human brains [81]. Phospho-tau protein is often present in tissue from different organs demonstrating amyloid pathology, including breast, heart, kidney, lung, muscle, testis, pancreas, skin, and fibroblasts [95,96,97].

Tau associated with amyloid deposits differs from normal tau in that it is hyperphosphorylated. This tau alteration decreases its capacity to bind to microtubules, compromises microtubule stabilization and axonal transport, and increases tau self-assembly and aggregate formation [81,98]. Phosphorylated tau, or phospo-tau, accompanies amyloid aggregate formation in many degenerative human conditions including cutaneous amyloidosis [85,86,95,96,99], and is expressed in organs along with curli fibers and neuropil threads [32]. In AD brains, hyperphosphorylated tau accumulates in pathological inclusions [80], and twisted fibrils present as either paired helical filaments or related straight filaments [81]. Neurofibrillary tangles are formed in neuronal cell bodies, while threads are formed in dendrites or axons [81]. Phosphorylation of tau protein has been shown to trigger tau protein aggregation into filamentous structures [100]. Tau proteins convert from an inert shape to a misfolded shape that can “seed” the growth of fibers. The misfolded “seed” triggers self-assembly of fibers that then stick together into insoluble aggregates [80].

Bb in vivo biofilms may be related to tau pathology. Exposure of primary neuronal and glial cells and brain cells to Bb induces tau phosphorylation in vitro [83]. In our study, MD skin sections were tested for the presence of phosho-tau, and all MD callus specimens stained positively, co-localizing with Bb/Hp biofilm structures and amyloid deposits. In addition, two specimens containing filamentous inclusions characteristic of MD stained positively for phospho-tau, Bb and Hp. Although MD fibers can contain collagen, keratin or both proteins, phospho-tau may be a component of some MD fibers. We hypothesize that tau protein may play a role in MD fiber formation by triggering protein aggregation into filamentous structures within MD skin. Bb/Hp positive aggregates stained positively for Thioflavin S, anti-β-amyloid protein and anti-phospho-tau, providing supportive evidence for the presence of amyloid structures within the mixed biofilms.

Although we have demonstrated biofilms containing Bb and Hp organisms in MD lesions, it is unclear whether these biofilms are responsible for the dermopathy found in MD. The fact that biofilms are associated with chronic bacterial infections and that a similar infectious dermopathy is seen in cattle and dogs argues that the biofilms containing Bb and Hp have a primary role in the skin pathology. Taken as a whole, our culture, histological and molecular findings are consistent with the modified Koch’s postulates that support a biofilm-related infectious etiology of MD lesions [101,102]. Additional pathogens may also be involved in formation of these biofilms [103]. Further studies in experimental animal models are needed to confirm the role of biofilms in the pathology of MD.

## 5. Conclusions

In summary, our study demonstrates dual infection with Bb and Hp in MD skin sections using tissue culture, IHC reactivity, PCR analysis, FISH testing and confocal microscopy. Overlapping IHC, PCR and FISH evaluation showed that Bb and Hp co-localize within aggregates exhibiting the biofilm-specific mucopolysaccharide alginate. Positive histochemical staining with Thioflavin S and positive IHC staining specific for β-amyloid and phospo-tau localized with the foci of Bb/Hp biofilm aggregates, providing evidence that amyloid and/or tau deposition could play a role in the establishment of biofilms and the subsequent evolution of MD lesions. Confocal microscopy revealed that Bb/Hp-positive aggregates were spatially distributed in a species-specific manner within biofilm structures, with Bb occupying the center and Hp occupying the periphery. The role of Bb/Hp biofilm aggregates in MD lesions merits further study.

## Figures and Tables

**Table 1:** PCR primers for *Borrelia burgdorferi* (Bb).

Gene Name	Gene Product	Location	Sequence (5′–3′)	Ref.
*pyrG*	CTP synthetase	Chromosome	Outer primers:	Margos et al., 2008 [25]
			F: ATTGCAAGTTCTGAGAATA	
			R: CAAACATTACGAGCAAATTC	
			Inner primers:	
			F: GATATGGAAAATATTTTATTTATTG	
			R: AAACCAAGACAAATTCCAAG	
*uvrA*	Exonuclease ABC; subunit A	Chromosome	Outer primers:	Margos et al., 2008 [25]
			F: GAAATTTTAAAGGAAATTAAAAGTAG	
			R: CAAGGAACAAAAACATCTGG	
			Inner primers:	
			F: GCTTAAATTTTTAATTGATGTTGG	
			R: CCTATTGGTTTTTGATTTATTTG	
*fla*	Flagellin	Chromosome	Outer primers:	Clark et al., 2013 [27]
			F: GCAGTTCARTCAGGTAACGG	
			R: TAGCAAGTGATGTATTRGCATCAA	
			Inner primers:	
			F: ACATATTCAGATGCAGACAGAGG	
			R: GAAGGTGCTGTAGCAGGTGCTGGC	
*ospC*	Outer surface protein C	Plasmid 26	Outer primers:	Wang et al., 1999 [24]
			F: AAAGAATACATTAAGTGCGATATT	
			R: GGGCTTGTAAGCTCTTTAACTG	
			Inner primers:	Bandoski and Sapi, 2013 [26]
			F: AAAGAATACATTAAGTGCGATATT	
			R: CTTGTAAGCTCTTTAACTGAATTAGC	

**Table 2:** PCR primers for *Helicobacter pylori* (Hp).

Gene Name	Gene Product	Location	Sequence (5′–3′)	Reference
16S rRNA	16S rRNA	Chromosome	Outer primers:	Karagin et al., 2010 [31]
			F: CTATGACGGGTATCCGGC	
			R: CTCACGACACGAGCTGAC	
			Inner primers:	
			F: CTATGACGGGTATCCGGC	
			R: TCGCCTTCGCAATGAGTATT	
*hsp60*	GroEL	Chromosome	Outer primers:	Singh et al., 2008 [30]
			F:AAGGCATGCAATTTGATAGAGGCT	
			R: CTTTTTTCTCTTTCATTTCCACTT	
			Inner primers:	
			F: TTGATAGAGGCTACCTCTCC	
			R: TGTCATAATCGCTTGTCGTGC	
*ureA*	Urease subunit α	Chromosome	Outer primers:	Donmez-Altuntas et al., 2002 [28]
			F: GCCAATGGTAAATTAGTT	
			R: CTCCTTAATTGTTTTTAC	
			Inner primers:	
			F: AGTTCCTGGTGAGTTGTTCT	
			R: AGCGCCATGAAAACCACGCT	
23S rRNA	23S rRNA	Chromosome	Outer primers:	Dewhist et al., 2005 [29]
			F: AGGCGATGAAGGACGTA	
			R: GGCCATGGATAGATCACT	
			Inner primers:	
			F: AGTAGYGGCGAGCGAA	
			R: AACTCGCAGGATCATTATG	

**Table 3:** Sequences of fluorescent in situ hybridization (FISH) probes for Bb and Hp.

Probe Name	Sequence 5′–3′	5′ Modification	Source
Borrelia 16S rDNA	GGATATAGTTAGAGATAATTATTCCCCGTTTG	6-FAM	Hammer et al., 2003 [33]
BbComp16s rDNA	CAAACGGGGAATAATTATCTCTAACTATATCC	None	Novel
Helicobacter 16S rDNA	TACCTCTCCCACACTCTAGAATAGTAGTTTCAAATGC	Alexa 568 dye	Camorlinga-Ponce, 2004 [34]; Liu et al., 2008 [35]
HpComp16s rDNA	GCATTTGAAACTACTATTCTGAGTGTGGGAGAGGTA	None	Novel
Random Probe	GCATAGCTCTATGACTCTATACTGGTACGTAG	6-FAM	Balasubramanian and Sapi, 2015 [36]

**Table 4:** PCR amplification of Bb and Hp target DNA, confirmed by sequencing.

Patient ID#	16S rRNA	Bb pyrG	Bb Fla	Bb uvrA	Bb ospC	Hp 16s rRNA	Hp 23s rRNA	Hp Hsp60	Hp Urea
1	−	+ f,r	+ f	−	+ f,r	+ f,r	−	−	+ r
2	−	+ f	−	−	−	+ f,r	+ f,r	+ f,r	+ f,r
3	+ f	+ f,r	+ f,r	+ f,r	+ f,r	+ f,r	−	+ f,r	-
4	−	+ f,r	−	−	−	+ f,r	−	−	−
5	+ f	+ f,r	−	−	−	+ f,r	−	+ f,r	+ r
6	−	−	−	+ f,r	−	+f,r	+f,r	+f	−
7	−	+ f,r	−	+ f	−	+ f,r	−	+ f,r	+ f,r
8	−	−	+ f	−	−	−	−	+ f,r	−
9	−	+ f	+ f	−	−	−	−	−	−
10	−	+f,r	−	−	−	−	−	−	−
11	−	−	−	−	−	+ f,r	−	−	−
12	−	−	−	−	−	+ f,r	−	−	−
13	−	−	−	−	−	+ f,r	+ f,r	+ f,r	+ f
14	−	−	−	−	−	−	+ f,r	−	+ f,r
Sample type									
Purchased skin	−	−	−	−	−	−	−	−	−
Normal skin, Morgellons subject	−	−	−	−	−	−	−	−	−
Normal skin, Lyme subjects	−	−	−	−	−	−	−	−	−
Normal skin, Healthy subjects	−	−	−	−	−	−	−	−	−

**Table 5:** Basic Local Alignment Search Tool (BLAST) analyses of Bb sequences.

Patient ID#	Amplicon	Closest Match %	E-Value	Query Cover
1	pyrG f 686bp	99% Bbss	0	96%
1	pyrG r 686bp	99% Bbss	0	97%
1	fla f 561bp	99% Bbss	3e−177	84–85%
1	ospC f 567bp	99% Bbss	0	86%
1	ospC r 568bp	99% Bbss	0	86%
2	pyrG f 682bp	99% Bbss	0	96%
3	16S f 397bp	99% Bbss	0	96%
3	pyrG f 682bp	100% Bbss	0	94%
3	fla f 409bp	99% Bbss	0	93%
3	fla r 566%	99% Bbss	0	93%
3	uvrA f 638	97% Bbss	0	97%
3	uvrA r 648bp	99% Bbss	0	97%
3	ospC f 560bp	99% Bbss	0	92%
3	osp C r 565bp	99% Bbss	0	91%
4	pyrG f 682bp	99% Bbss	0	97%
4	pyrG r 682 bp	99% Bbss	0	97%
5	16S f 473bp	99% Bbss	0	76%
5	pyrG f 747	99% Bbss	0	86%
5	pyrG r 685	100% Bbss	0	94%
6	uvrA f 653bp	99% Bbss	0	96%
6	uvrA r 651	99% Bbss	0	99%
7	pyrG f 674	100% Bbss	0	94%
7	pyrG r 684	100% Bbss	0	94%
7	uvrA f 486	96% Bbss	0	93%
8	fla 663 f bp	100% Bbss	4e−177	100%
9	pyrG f 680bp	100% Bbss	0	94%
9	fla f 367bp	100% Bbss	2e−172	90%
10	pyrG f 682bp	99% Bbss	0	97%
10	pyrG r 682 bp	99%Bbss	0	97%
11	NONE			
12	NONE			
13	NONE			
14	NONE			

**Table 6:** BLAST analyses of Hp sequences.

Patient ID#	Amplicon	Closest Match %	E-Value	Query Cover
1	16S f 406 bp	99% *H. pylori*	0	95%
1	16S r 407 bp	99% *H. pylori*	0	94%
1	ureA r 339 bp	99% *H. pylori*	1e−140	83%
2	16S f 407 bp	100% *H. canis*	0	95%
2	16S r 409 bp	100% *H. canis*	0	95%
2	23S f 359 bp	99% *H pylori*	5e−174	95%
2	23S r 358 bp	99% *H. pylori*	5e−174	94%
2	hsp60 f 472 bp	99% *H. pylori*	0	95%
2	hsp60 r 476 bp	99% *H. pylori*	0	93%
2	ureA f 337 bp	99% *H. pylori*	3e−151	89%
2	ureA r 333 bp	99% *H. pylori*	2e−157	93%
3	16S f 403 bp	99% *Wolinella succinogenes* (in family *Helicobacteracea*)	0	94%
3	16S r 408 bp	99 % *W. succinogenes*	0	94%
3	Hsp60 f 891 bp	100% *H. pylori*	0	50%
3	Hsp60 f 678 bp	99% *H. pylori*	0	90%
3	Hsp60 r 470 bp	99% *H pylori*	0	96%
4	16S f 377 bp	99% *H. canis*	2e−167	87%
4	16S r 411 bp	99% *H. canis*	0	90%
5	16S f 401 bp	99% *H. pylori*	0	94%
5	16S r 410 bp	100% *H. pylori*	0	92%
5	ureA 557 bp	99% *H. pylori*	7e−164	57%
5	Hsp60 f 729 bp	99% *H. pylori*	0	96%
5	Hsp60 r475 bp	99% *H. pylori*	0	96%
6	16S f 421 bp	100% *H. canis*	0	88%
6	16S r 413 bp	100% *H. canis*	0	90%
6	23S f 356 bp	99% *H. pylori*	2e−172	94%
6	23S r 358bp	99% *H pylori*	6e−178	96%
6	Hsp60 f 434 bp	88% *H. pylori*	1e−145	93%
7	16S f 375 bp	99% *H. pylori*	1e−175	92%
7	16S r 408 bp	99% *H. pylori*	0	91%
7	Hsp60 f 474 bp	99% *H. pylori*	0	93%
7	Hsp60 r 483 bp	97% *H. pylori*	0	93%
7	urea f 337 bp	99% *H. pylori*	3e−151	89%
7	urea r 333 bp	99% *H. pylori*	2e−1157	93%
8	Hsp60 474 bp	99% *H. pylori*	0	94%
8	Hsp60 483 bp	100% *H. pylori*	0	90%
9	NONE			
10	NONE			
11	16S f 373 bp	99% *H. pylori*	2e−172	90%
11	16S r 412 bp	99% *H. pylori*	0	91%
12	16S f 408 bp	99% *H. canis*	0	96%
12	16S r 408 bp	99% *H. canis*	0	94%
13	16S f 366 bp	97% *H. pylori*	4e−165	96%
13	16S r 403 bp	97% *H. pylori*	0	93%
13	23S f 558 bp	96% *H. pylori*	3e−138	70%
13	23S r 599 bp	97% *H. pylori*	0	86%
13	Hsp60 732 bp	99% *H. pylori*	0	79%
13	Hsp60 562 bp	100% *H. pylori*	0	80%
13	urea 332 bp	99% *H. pylori*	5e−1158	93%
14	23S f 358 bp	99% *Arcobacter butzerli*	7e−177	96%
14	23S r 359 bp	99% *Arcobacter butzerli*	1e−174	95%
14	ureA f329 bp	91% *H. pylori*	2e−92	76%
14	ureA r	76% *H. pylori*	1e−31	48%

**Table 7:** Summary of histochemical staining. NP, not performed.

Patient ID#	Antibody			Bb FISH	Hp FISH	Anti-β Amyloid		Phospho-tau	Thioflavin
	Anti-Bb	Anti-Hp	Anti-Alginate			Antibody 1	Antibody 2		
1	+	NP	+	+ (not overlapping)	+ (not overlapping)	−	+	+	+
2	+	+	+	+	+	−	+	+	NP
3	+	+	+	+	+	−	+	+	NP
4	+	+	+	+	+	−	+	+	NP
5	+	+	+	+	+	−	+	+	NP
6	+	+	+	+	+	−	+	+	+
